# A deep learning algorithm for automatic 3D segmentation and classification of the sheep placenta in magnetic resonance images

**DOI:** 10.14814/phy2.70869

**Published:** 2026-04-17

**Authors:** Dimitra Flouri, Giorgos Adamides, Jack R. T. Darby, Stacey L. Holman, Andreas S. Panayides, Anna L. David, Vasileios Vavourakis, Janna L. Morrison, Andrew Melbourne

**Affiliations:** ^1^ In Silico Modelling Group, Department of Mechanical and Manufacturing Engineering University of Cyprus Nicosia Cyprus; ^2^ School of Biomedical Engineering and Imaging Sciences King's College London London UK; ^3^ 3AE Health LTD Nicosia Cyprus; ^4^ Early Origins of Adult Health Research Group, School of Pharmacy and Biomedical Sciences, Robinson Research Institute, College of Health Adelaide University Adelaide South Australia Australia; ^5^ CYENS Centre of Excellence Nicosia Cyprus; ^6^ Elizabeth Garrett Anderson Institute for Women's Health University College London London UK; ^7^ Department of Medical Physics and Biomedical Engineering University College London London UK

**Keywords:** convolutional neural networks, deep learning, magnetic resonance imaging, placenta, sheep

## Abstract

Magnetic resonance imaging (MRI) is a powerful non‐invasive method for assessing placental morphology and physiology in vivo, offering higher resolution and functional insight than ultrasound. However, placental MRI analysis is limited by manual segmentation and time‐consuming classification workflows that are prone to inter‐operator variability in preclinical sheep models. This study presents the first fully automated MRI analysis pipeline for the ovine placenta, integrating advanced deep learning models to accelerate and standardize preclinical placental assessment. We evaluated 2D and 3D configurations of the self‐configuring nnU‐Net against a conventional 2D U‐Net, training on expert‐annotated datasets and testing on a held‐out set. The 3D nnU‐Net achieved a Dice score above 0.81, outperforming both 2D approaches while reducing processing time by more than 95% and closely matching manual contours. Using these segmentations, we implemented a YOLOv11‐based detection module to localize and classify individual placentomes into four morphological types (A–D), achieving 88.7% precision, 92.1% recall, mAP50 of 95.1%, and mAP50‐95 of 93.5%. This dual‐stage workflow enables fully automated, in vivo quantification of placentome morphology in sheep, supporting high‐throughput, reproducible assessment of placental structural and functional adaptation while reducing operator bias and manual burden, and laying the foundation for MRI biomarker validation.

## 1 INTRODUCTION

The placenta is critical for fetal growth and development, facilitating the transfer of oxygen and nutrients from mother to fetus. Abnormalities in placental development and function underlie many pathologies of pregnancy, including preeclampsia, fetal growth restriction, and spontaneous preterm birth (Liu et al., 2022; Preston et al., 2024). Placenta volume is the only known morphological factor that can predict adverse perinatal outcomes (Manokhina et al., 2017). A small placenta in the first trimester is associated with an increased risk of reduced preeclampsia and fetal growth (Collins et al., 2013). A growing body of evidence also highlights the importance of placental dysfunction to the lifelong health of both the mother and fetus (Longtine & Nelson, 2011; Turco & Moffett, 2019). Therefore, placental assessment is crucial for understanding the placenta's structure, development, and function to identify strategies to optimize pregnancy outcomes.

Ultrasound is the primary imaging modality for monitoring fetal health and development (Kamankesh et al., 2020). However, it is susceptible to the position of the placenta, the amount of amniotic fluid, and the thickness of the abdominal wall adipose tissue of pregnant women, resulting in less accurate results (Munoz et al., 2019). Magnetic resonance imaging (MRI) is not affected by these factors and is an ionizing radiation‐free, non‐invasive imaging modality that provides high soft tissue resolution (Manganaro et al., 2018). Emerging MRI techniques increasingly provide robust information on placental function in vivo to support clinical decision‐making (Flouri et al., 2020; Melbourne et al., 2019; Saini et al., 2021). Assessment of novel placental imaging biomarkers for evaluating fetal health relies on the use of animal models. Placental imaging measurements are difficult to validate due to the invasive nature of gold‐standard procedures such as cordocentesis; thus, preclinical models are important for validation studies of MRI measurements, as they allow controlled experiments and analysis of multiple time points during pregnancy (Flouri et al., 2021; Morrison et al., 2018).

The pregnant sheep has been used extensively to investigate maternal‐fetal interactions and has provided an evidence base for the clinical translation of intervention strategies to improve pregnancy outcomes (Flouri et al., 2022; Flouri et al. 2025). In contrast to the human discoid placenta, the sheep placenta is comprised of multiple discrete placentomes (between 60 and 80) (Poudel et al., 2015). These placentomes are classified into four morphological types (A–D) according to their gross morphological appearance, which are thought to be related to vascularity and nutrient transfer in the pregnant ewe (Ward et al., 2006). The complexity and variety of placentomes make the development of automatic segmentation and classification algorithms more challenging.

Currently the assessment of placental function in sheep from MR images relies on the manual selection and slice‐by‐slice segmentation of numerous placentomes. As the number of animals in a preclinical study can be large with scans performed at multiple timepoints, this task can add hours of laborious analysis. In addition, the quality of the resulting segmentation depends on expert experience and often suffers from high intra‐ and inter‐operator variability. Therefore, fully automated segmentation techniques to standardize and accelerate the segmentation of sheep placentomes are highly desirable for placental MRI studies in pregnant sheep.

### 1.1 Prior work in placental MRI segmentation

Recognizing the limitations of manual contouring, a range of semi‐ and fully automated placental segmentation methods have been developed for human MRI. Early approaches focused on semi‐automatic frameworks combining classical machine‐learning techniques with user interaction, such as random forest classifiers integrated with conditional random fields (Wang et al., 2016; Wang et al., 2019). Subsequent studies adopted deep learning–based methods, including minimally interactive convolutional neural networks with region‐growing refinement (Shahedi et al., 2020) and fully automated 3D U‐Net architectures for placental and uterine cavity segmentation (Shahedi et al., 2021; Shahedi et al., 2022). More recent work has explored advanced network designs to address challenges such as motion artifacts and anatomical variability, including multi‐scale 3D convolutional neural networks (Alansary et al., 2016), spatial attention mechanisms (Liu et al., 2023), and refinement‐based or fusion U‐Net variants (Huang et al., 2023; Li et al., 2024). A structured comparison of these studies is provided in Table S1.

### 1.2 Study aims and contributions

Despite these advances, all existing methods have been developed for the human placenta, which is anatomically distinct from the ovine placenta. The sheep placenta comprises multiple discrete placentomes, each with unique morphology and variable appearance across gestation. No prior study has attempted automated segmentation of ovine placentomes or automatic classification of placentome morphological types from MRI data. This gap is significant, as preclinical sheep models are central to validating emerging placental imaging biomarkers and translating them into clinical practice.

The present work addresses this unmet need by introducing the first fully automated MRI workflow for analyzing sheep placentomes. Our approach performs automated segmentation of individual placentomes and classifies them into established morphological types (A–D). This study fills a critical gap in ovine placenta MRI and supports the development and validation of emerging MRI biomarkers of placental health.

## 2 METHODS

### 2.1 Ethics approval

The data collection and analysis followed relevant guidelines and regulations. The experimental protocols were reviewed and approved by the Animal Ethics Committee of the South Australian Health and Medical Research Institute (SAHMRI) and abide by the Australian Code of Practice for the Care and Use of Animals for Scientific Purposes (2013) developed by the National Health and Medical Research Council, and followed the ARRIVE guidelines (Percie du Sert et al., 2020). All investigators were familiar with the ethical principles outlined in Grundy (2015) and O'Halloran (2024) and adhered to the principles of the 3Rs (Steinmeyer et al., 1995).

### 2.2 Data acquisition and preparation

#### 2.2.1 Animal preparation

Merino ewes (*n* = 61) were sourced from the SAHMRI Farm (Burra, South Australia, Australia) and housed in individual pens in animal holding rooms at a constant ambient temperature of 20°C–22°C and a 12 h:12 h light–dark cycle with ad libitum access to food and water. A cohort of non‐pregnant ewes (*n* = 28) underwent surgical removal of the majority of their endometrial caruncles via carunclectomy (CX) (Danielson et al., 2005). Caruncles are specialized uterine structures that serve as sites for placental attachment; this intervention is known to induce intrauterine growth restriction in subsequent pregnancies (Flouri et al., 2022). After a minimum recovery period of 10 weeks, both CX ewes and Control ewes (*n* = 33), which had not undergone any prior surgical intervention, were enrolled in a mating program. Prior to MRI scanning, ewes fasted for at least 12 h and were positioned in left lateral recumbency. General anesthesia was induced via intubation and administration of diazepam (0.3 mg kg^−1^, ilium, Troy Laboratories Pty Ltd., New South Wales) and ketamine (5 mg kg^−1^, ilium, Ceva Animal Health Pty Ltd., New South Wales) and maintained with 2.0%–3.0% isoflurane (Lyppards) (Varcoe et al., 2019). Maternal heart rate and arterial oxygen saturation were continuously monitored using an MRI‐compatible pulse oximeter (Nonin Medical Inc., Plymouth, MN, USA) and recorded via LabChart 7 (AD Instruments, Castle Hill, Australia). The ewes were ventilated at a respiratory rate of 16–18 breaths min^−1^ and 1.0–3.0 L of oxygen was titrated with 3.0–5.0 L of air.

#### 2.2.2 MRI acquisition

A placenta‐specific imaging methodology mechanism was employed (Melbourne et al., 2019) that combines Diffusion‐Weighted Imaging (DWI) and T2‐relaxometry. DWI was performed at ten *b*‐values: *b* = [0, 10, 20, 30, 50, 70, 100, 200, 300, 500, 600] $s.{\mathrm{mm}}^{-2}$, and echo‐time (T_E_) = 72 ms. Spin‐echo T2‐relaxometry was acquired at *b*‐value = 0 $s.{\mathrm{mm}}^{-2}$ and at ten echo times: T_E_ = [81, 90, 96, 120, 150, 180, 210, 240, 270, 300] ms. Data were acquired at *b*‐values 50 and 200 for T_E_ = [81, 90, 120, 150, 180, 210, 240] ms. The data were acquired with a pulse gradient spin‐echo with EPI readout sensitive to diffusion in the slice plane with the following parameters: flip angle = 90°, voxel size 0.9 × 0.9 × 6 mm^3^, with field of view 350 × 281 mm^2^ and 26 slices.

#### 2.2.3 Image database

In this retrospective, single‐centre study, we assembled a database of sheep placental MRI examinations acquired between 2019 and 2025 on a 3 T Siemens Skyra MRI scanner (Siemens Healthcare, Erlangen, Germany). Only singleton pregnant ewes were included. The dataset comprised 61 pregnant ewes, including 33 Control ewes (61 MRI examinations) and 28 CX ewes (45 MRI examinations). MRI examinations were performed at three gestational ages: early gestation (80 days; *n* = 5 examinations), mid‐gestation (109–111 days; *n* = 87 examinations), and late gestation (139–141 days; *n* = 14 examinations) (Cho et al., 2024; Darby et al., 2024; Duan et al., 2019; Saini et al., 2021). In total, 106 volumetric MRI examinations were acquired across the 61 animals, reflecting repeated imaging of some animals at multiple gestational timepoints. Across all volumetric MRI examinations, a total of 1740 placentomes were manually annotated and used for model training and evaluation. For model development, data were partitioned at the animal level to prevent data leakage. Fifty animals (89 volumetric MRI examinations; 52 Controls, 37 CXs) were allocated to training and cross‐validation, and eleven animals (17 volumetric MRI examinations; 11 Controls, 8 CXs) were reserved for independent testing. No MRI volumes from the same animal appeared in both training and test sets.

### 2.3 Definition of gold standard

The placentome regions of interest (ROI) were manually segmented from the first *b* = 0 image (ITK‐SNAP Version 4.2.2, 2024) by a single experienced operator, following established placentome delineation criteria (Flouri et al., 2021) to ensure consistency across the dataset. These manual annotations are referred to hereafter as the ‘gold standard’ for model training and evaluation purposes. Although placentome segmentation is a challenging and potentially subjective task, inter‐observer variability for placentome morphological classification has been previously assessed in a representative subset of this cohort (Flouri et al., 2021), demonstrating high overall agreement between independent operators.

To avoid inclusion of non‐placentome tissue, the ROIs were positioned away from the placentome edges so that residual motion artifacts from maternal breathing or fetal movement would not displace the ROI outside the placentome area. Each ROI was defined to cover the largest area common to all timepoints of the dynamic acquisition. Rigid registration (Klein et al., 2010) followed by non‐rigid free‐form registration (Flouri et al., 2020) was applied to reduce motion artifacts. Placental MRI was used to morphologically classify placentomes into four types (A–D) (Flouri et al., 2021). In normal pregnancy, type A and B placentomes are predominant, whereas types C and D placentomes are less common.

### 2.4 Automatic placentome segmentation

To achieve automated segmentation, we trained and compared conventional U‐Net (Ronneberger et al., 2015) and Attention U‐Net (attU‐Net) architectures (Oktay et al., 2018), as well as the self‐configuring nnU‐Net framework in both 2D and 3D configurations, using manually segmented image stacks as the gold standard. These models were selected to represent a progression from baseline convolutional encoder‐decoder networks (U‐Net), through established attention‐augmented variants (attU‐Net), to a widely adopted, self‐configuring segmentation framework (nnU‐Net) that is commonly used as a benchmark reference in medical imaging studies (Isensee et al., 2021). This comparative design allowed us to systematically evaluate how increasing model complexity (from 2D slice‐based to volumetric 3D representations) affect segmentation performance in placentome MRI, where structures are small, discrete and spatially heterogeneous. The U‐Net and attU‐Net models were implemented in Python v3.10 with TensorFlow v2.5.0 and trained on a desktop workstation equipped with an NVIDIA GeForce 4090 RTX GPU (24 GB VRAM). The 2D and 3D nnU‐Net models were trained using the framework's native PyTorch implementation (v2.2.2 with CUDA 12.1) on a high‐performance computing (HPC) cluster provided by the University of Cyprus. Each GPU node was a Dell C4140 equipped with Intel Xeon 6240 CPUs (2.6 GHz, 18 cores), 384 GB RAM, and four NVIDIA Tesla V100 GPUs (32 GB SXM2 each). A single V100 GPU was used per training run.

#### 2.4.1 Dataset partitioning and stratified sampling

To achieve a comprehensive and balanced model training and evaluation process, dataset partitioning was performed at the individual animal level to prevent data leakage across scans from the same subject. An 80/20 split was applied, with 80% of animals assigned to model development (training and validation) and 20% reserved as an independent test set. Animals were categorized into “CX” and “Control” groups based on predefined identifiers, and stratified sampling was applied within each group to preserve class balance. The resulting training and testing cohorts were formed by merging the corresponding CX and Control subsets, ensuring that both datasets maintained a balanced representation of clinical status, thereby supporting generalizable model development. The independent test set (*n* = 11 animals) was held out entirely and never used during training or validation for any model. All final performance metrics reported in this study were computed exclusively on this same independent test set, enabling fair and direct comparison across models.

#### 2.4.2 Image preprocessing for 2D models

Prior to training the 2D U‐Net and Attention U‐Net models, the MRI data underwent a dedicated preprocessing pipeline. This preprocessing was applied exclusively to the 2D models, as the nnU‐Net framework performs its own automated intensity normalization and preprocessing. All input images were resampled via interpolation to a standard in‐plane resolution of 256 × 256 pixels to ensure consistent spatial dimensions across the dataset.

To mitigate the influence of extremely large pixel values in the raw MRI data, an upper intensity threshold of 2500 was applied prior to normalization, preventing outlier intensities from negatively influencing the normalization process. Image normalization was performed by scaling pixel values to a uniform range, reducing the impact of intensity variability. The normalized MRIs were scaled from 0 (black) to 255 (white), using the following intensity normalization function:

(1) $$f\left(x,y\right)=\frac{\mathrm{GWM}-\mathrm{BWM}}{g_{\max}-g_{\min}}\times \left(g\left(x,y\right)-g_{\min}\right)+\mathrm{BWM}$$

where 𝑓(𝑥, 𝑦) represents the normalized pixel value, 𝑔(𝑥, 𝑦) denotes the original pixel value, $g_{\max}$ and $g_{\min}$ correspond to the maximum and minimum intensities identified in the histogram, and 𝐺𝑊𝑀 and 𝐵𝑊𝑀 were set to 255 and 0, respectively.

#### 2.4.3 2D U‐net and AttU‐net architectures

The dataset was partitioned at the animal level using a fixed 70/10/20 split, with 70% of animals used for training, 10% for validation, and 20% held out as an independent test set, as described above. The 80% development set (training + validation) was used to optimize the 2D U‐Net and attU‐Net models, preserving the animal‐level split and preventing scan‐level leakage. Both architectures were initialized with ImageNet‐pretrained encoder weights (Deng et al., 2009) following standard transfer‐learning practice. Models were trained for up to 100 epochs; however, due to the use of pretrained weights, convergence was reached substantially earlier, and final model weights were selected based on stable validation performance prior to the onset of training–validation divergence. The use of transfer learning facilitated faster convergence, reduced the number of epochs required for optimal performance, and lowered computational demand compared with training from scratch.

#### 2.4.4 nnU‐net framework, cross‐validation, and model optimization

**FIGURE 1:**
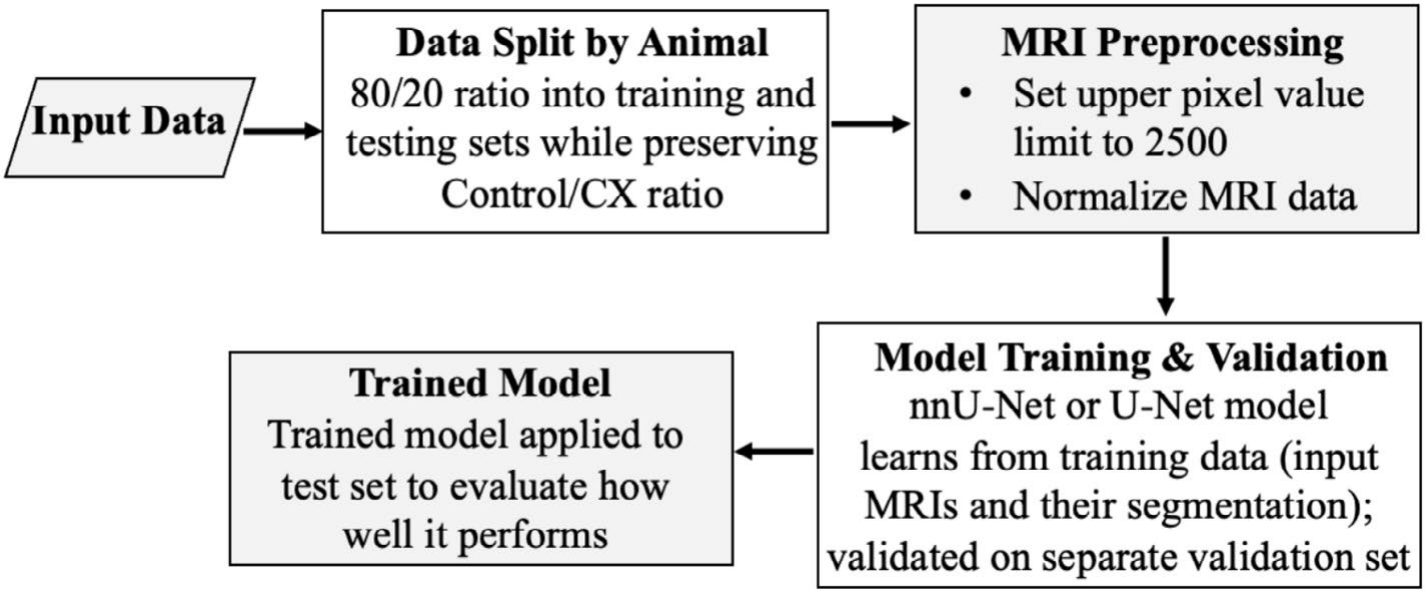
Schematic overview of the MRI preprocessing and segmentation pipeline.

The nnU‐Net framework (Isensee et al., 2024) was employed to perform both 2D and 3D image segmentation. As illustrated in Figure 1, MRI data were first organized according to the standardized folder structure outlined in the nnU‐Net documentation, ensuring full compatibility with the framework's automated preprocessing pipeline. In contrast to conventional 2D U‐Net and attU‐Net architectures, the nnU‐Net framework performs intensity normalization during preprocessing, improving consistency across all MRI scans.

For model optimization, nnU‐Net was configured with its recommended 5‐fold crossvalidation scheme on the same 80% development set described above. The independent 20% test set was excluded from all folds and used only for final evaluation. The development set was randomly divided into five folds; in each iteration, one fold served as the validation set while the remaining four folds were used for training. This process was repeated five times so that every fold acted as the validation set once, ensuring comprehensive model assessment across the training data. All folds were trained for up to 1000 epochs using stochastic gradient descent with nnU‐Net's built‐in early stopping and learning‐rate scheduling mechanisms to maximize convergence.

Unlike the 2D U‐Net and attU‐Net models, which were initialised with ImageNet‐pretrained encoders and therefore reached convergence rapidly, nnU‐Net trains its architectures from scratch. This inherently requires a much longer training schedule (up to 1000 epochs) and accounts for the difference in training duration across models.

#### 2.4.5 Model evaluation

Segmentation performance was evaluated using four metrics: the Dice similarity coefficient (DSC), Precision, Recall (sensitivity), and the 95th percentile Hausdorff distance (HD95), all computed according to established definitions described by Taha and Hanbury (2015). The DSC quantified spatial overlap between predicted segmentation masks and the manually annotated gold standard, with values ranging from 0 (i.e., no overlap) to 1 (i.e., perfect agreement). Precision measured the proportion of predicted placentome voxels that were correctly classified, while Recall quantified the proportion of true placentome voxels successfully identified by the model. Boundary agreement was assessed using HD95, which measures the 95th percentile of the surface distance between the predicted and reference segmentations, thereby reducing sensitivity to outlier errors.

### 2.5 Automatic placentome classification

In this study, we used the YOLOv11‐X object detection model to classify sheep placentomes from MRI scans (Khanam & Hussain, 2024). YOLOv11‐X is a single‐stage detector, meaning it identifies all placentomes and predicts their types in one step, allowing efficient analysis of MRI scans that contain many structures. Although YOLOv11‐X jointly performs object localisation and class prediction, it operates on 2D images and produces bounding‐box detections rather than voxel‐wise segmentation masks. Consequently, it cannot provide the precise 3D placentome delineation required for volumetric quantification. For this reason, segmentation and classification were treated as complementary but distinct tasks: volumetric placentome segmentation was performed using 3D nnU‐Net, while YOLOv11‐X was applied independently to 2D MRI slices for placentome localisation and morphological classification.

As the largest and most accurate version of the YOLOv11 family (Ali & Zhang, 2024), YOLOv11‐X employs a deep convolutional backbone with cross‐stage partial (CSP) connections, a feature pyramid/path aggregation neck for multi‐scale feature fusion, and multi‐scale detection heads for object classification and localisation. The X variant is obtained through increased depth and width scaling, resulting in approximately 70 million trainable parameters, substantially more than the lightweight YOLOv11 variants (e.g., S or M), which are optimized for real‐time performance (Khanam & Hussain, 2024). This increased model capacity enables improved detection of placentomes across a wide range of sizes and contrast levels, including structures with faint boundaries relative to surrounding uterine tissue. Because real‐time performance was not a requirement in this study and computational resources were not a limiting factor, we selected the YOLOv11‐X model rather than a lightweight version, which uses fewer parameters and is optimized for speed, potentially at the expense of detection accuracy.

Due to the limited number of placentomes in categories C and D, the placentomes were grouped into two broader categories: AB and CD. Because YOLOv11‐X processes 2D images, classification was performed on individual 2D MRI slices. The model first generated candidate bounding boxes with an associated confidence score indicating the likelihood that each box contained a placentome. For boxes passing the confidence threshold, the model then produced a probability distribution over the two classes (AB and CD), reflecting the confidence of the class assignment. For evaluation purposes, each detected placentome was classified according to the class with the highest predicted probability, resulting in a discrete classification. These probabilities can also be used to identify ambiguous cases where the model is uncertain between AB and CD. The classification models were evaluated on 442 MRI slices extracted from the 17 MRI volumes in the test set, containing a total of 1740 manually annotated placentome instances. To assess localisation accuracy, we adopted a stricter intersection‐over‐union (IoU) threshold of 0.75 to decide whether a predicted box truly corresponds to a placentome or should be treated as background. Predictions with IoU ≥0.75 relative to a gold standard placentome were counted as a true detection; predictions with IoU <0.75 (including those overlapping only marginally) were treated as false positives against the background. Likewise, any gold standard placentome without a matching prediction at IoU ≥0.75 was recorded as a false negative. A confidence threshold of 0.5 was applied throughout evaluation.

Classification performance was summarized using a normalized confusion matrix across three classes: AB placentomes, CD placentomes, and background. The background class was included to represent non‐placentome regions of the image. This class covers regions where the model incorrectly detected a placentome even though none were present (false positives), as well as areas correctly identified as empty. Each column of the matrix was normalized to sum to 1, allowing diagonal entries to represent per‐class recall and off‐diagonal entries to reflect misclassification patterns.

#### 2.5.1 Evaluation metrics

Model performance in classifying placentomes was assessed using the Precision, Recall, and F1 score metrics for each class (AB placentomes, CD placentomes, and Background). Precision quantified the proportion of detected placentomes that were correctly classified, while Recall measured the proportion of true placentomes successfully identified by the model. The F1 score was used as a harmonic mean of Precision and Recall, providing a balanced measure of classification performance.

### 2.6 Statistical analysis

All statistical analyses were performed on the testing group to evaluate biological differences in placentome morphology and assess segmentation accuracy. Linear regression analysis was performed between the predicted and manually derived (gold standard) placentome volumes for each model (2D nnU‐Net, 3D nnU‐Net, U‐Net and attU‐Net). The coefficient of determination (R^2^), Pearson correlation coefficient (r), and regression equations were calculated. Agreement between predicted and gold standard measurements was further assessed using Bland–Altman analysis, with bias and 95% limits of agreement calculated as mean difference ± 1.96 × standard deviation (SD). Volumetric data were aggregated per animal and placentome type prior to statistical analysis. Placentome volumes for Types A and B were analyzed using a two‐way repeated‐measures ANOVA, with type (A vs. B) as the within‐subject factor and Group (Control vs. CX) as the between‐subject factor. Only Types A and B were included in the repeated‐measures ANOVA, as Type D placentomes were absent in the Control group and Type C placentomes were limited in number (*n* = 3), preventing reliable statistical comparison. Normality of volume distributions was evaluated using the Shapiro–Wilk test. All statistical tests were performed in MATLAB 2024b (The MathWorks, Natick, MA, USA). Statistical significance was defined as *p* < 0.05.

## 3 RESULTS

### 3.1 Placentome segmentation

**FIGURE 2:**
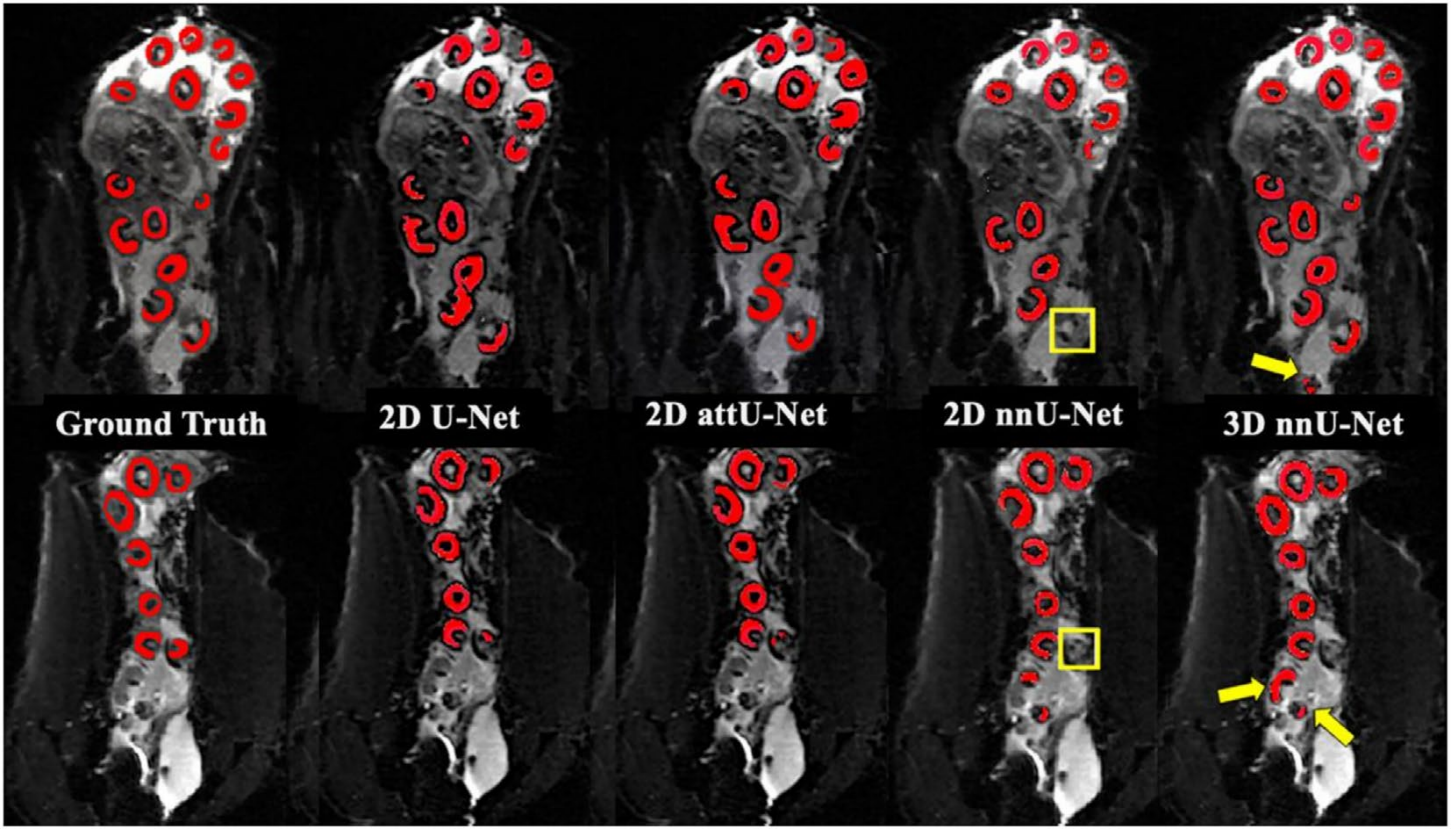
Qualitative comparison of placentome segmentation performance across models. Representative sagittal MRI slices are shown with red outlines denoting segmented placentomes. From left to right in each row: Manual gold standard contours, 2D U‐Net predictions, 2D attU‐Net predictions, 2D nnU‐Net predictions, and 3D nnU‐Net predictions. Yellow arrows highlight regions where the 3D nnU‐Net detects placentomes that were not detected in the gold standard (false positives), and yellow boxes indicate placentomes missed by the 2D U‐Net (false negatives).

Representative segmentation results obtained with U‐Net, attU‐Net, 2D nnU‐Net, and 3D nnU‐Net are shown in Figure 2. Qualitative analysis indicates that the 3D nnU‐Net segmentation outputs exhibit the highest correspondence with manual annotations, accurately delineating placentomes across the volume. Notably, 3D nnU‐Net also detected additional candidate placentomes not included in the manual gold standard annotations (see arrows in Figure 2). These regions were subsequently re‐reviewed by the original annotator, who confirmed that 5 corresponded to true placentomes that had been missed during the original manual labeling, while 4 represented false positives. This highlights the model's ability to detect subtle anatomical structures, as well as the limitations of manual annotation, which may miss small or ambiguous placentomes. In contrast, the 2D‐based models show characteristic shortcomings. Both U‐Net and attU‐Net suffer from slice‐by‐slice discontinuities, leading to fragmented or incomplete segmentations across adjacent slices. The attU‐Net slightly improves boundary delineation compared to U‐Net but still struggles with atypical placentome morphologies and those located near image borders. The 2D nnU‐Net shows better robustness than the U‐Net and attU‐Net baseline models but nevertheless fails to capture certain placentomes, particularly in challenging regions (see boxes in Figure 2). The U‐Net demonstrates both under‐ and over‐segmentation artifacts in these challenging areas. This suggests that 2D segmentation approaches may be less effective in preserving spatial continuity and structural coherence across slices.

Table 1 summarizes the segmentation performance on the test set for the four network configurations applied to automatic sheep placentome segmentation. The 3D nnU‐Net achieved the highest DSC of 0.81, followed by the 2D nnU‐Net with a DSC of 0.74, while the U‐Net and attU‐Net plateaued at lower DSC values of 0.63 and 0.64, respectively. Both nnU‐Net models were trained from scratch for 1000 epochs to ensure sufficient convergence. In contrast, the 2D U‐Net and attU‐Net models were initialized with pretrained weights and trained for 100 epochs. Extending training beyond this point did not yield further improvements, with final DSCs plateauing at 0.62 and 0.63, respectively. Boundary accuracy, assessed using the 95th percentile Hausdorff distance (HD95), exhibited a similar trend. The 3D nnU‐Net achieved the lowest HD95 (1.89 mm), markedly lower than the 2D nnU‐Net (3.75 mm) and the U‐Net and AttU‐Net baselines (>12 mm). This demonstrates superior boundary accuracy and spatial consistency for the 3D model.

**TABLE 1:** Quantitative segmentation performance on the test set for the four network configurations. Reported metrics include training epochs, dice similarity coefficient (DSC), precision, recall, and 95th percentile Hausdorff distance (HD95).

Model	Epochs	DSC	Precision	Recall	HD95 (mm)
2D U‐Net	100	0.62	0.56	0.71	16.29
2D attU‐Net	100	0.63	0.56	0.77	12.05
2D nnU‐Net	1000	0.74	0.85	0.67	3.75
3D nnU‐Net	1000	0.81	0.86	0.79	1.89

In addition to DSC, the 3D nnU‐Net also achieved the lowest 95th percentile Hausdorff distance (HD95) of 1.89 mm, indicating high boundary accuracy and spatial consistency. Precision and recall metrics further highlight the model's robustness: the 3D nnU‐Net balanced both metrics well (precision: 0.86, recall: 0.79), whereas the 2D nnU‐Net showed high precision (0.85) but low recall (0.67), suggesting it was more conservative in its predictions. The U‐Net and attU‐Net models exhibited lower precision and recall overall.

**FIGURE 3:**
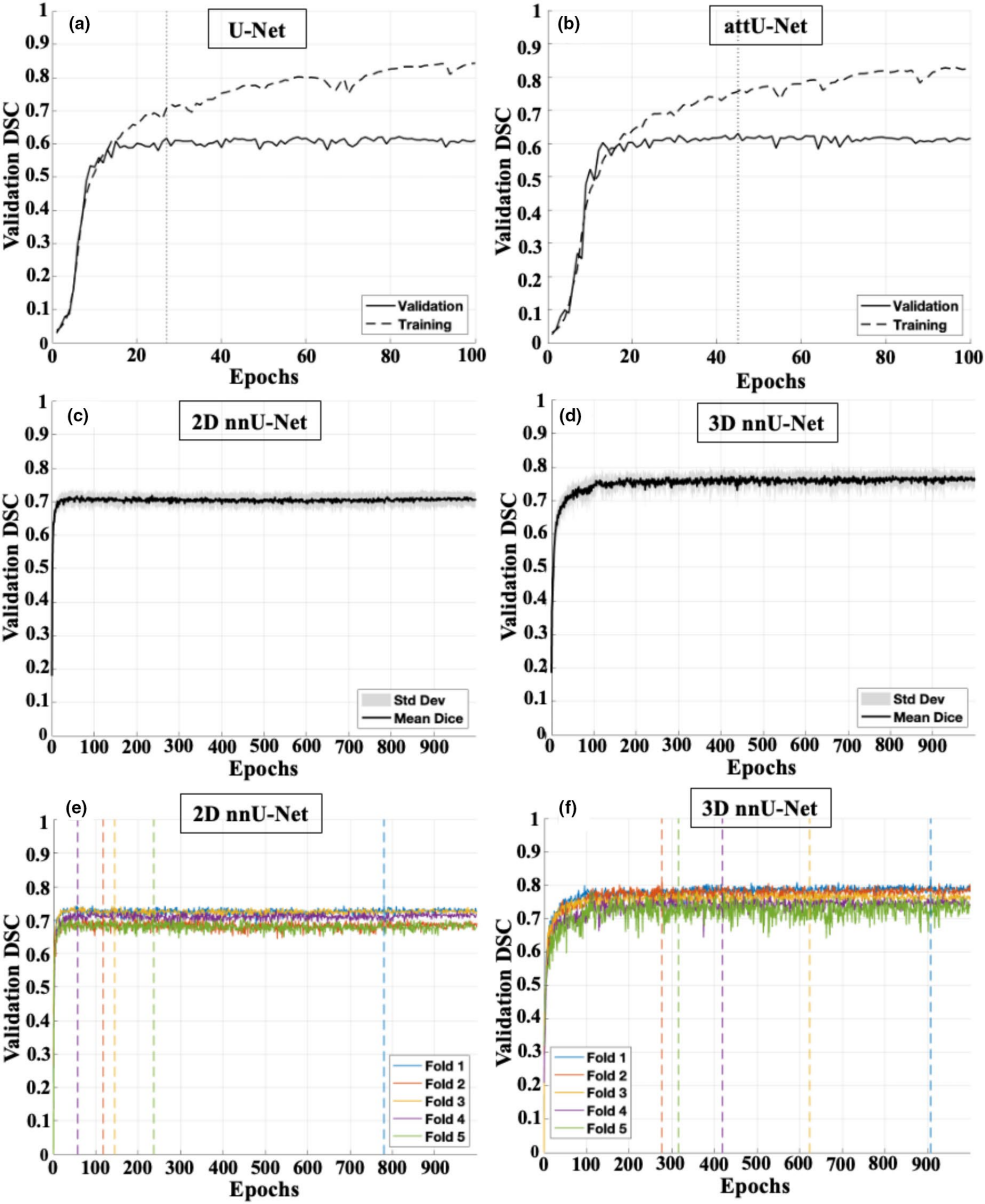
Performance comparison of four neural network models for automatic segmentation of sheep placentomes. (a, b) Training (dashed line) and validation (solid line) dice similarity coefficient (DSC), curves over epochs for U‐Net and attU‐Net. Vertical dashed lines indicate the epoch at which the highest validation DSC was recorded. (c, d): Mean validation DSC (solid line) across 5 folds for 2D and 3D nnU‐Net, with shaded area representing ± standard deviation. (e, f): Training curves of 2D and 3D nnU‐Net models across five cross‐validation folds. Line plots show the DSC on the validation set over 900 train 1000 training epochs for each fold. The 3D nnU‐Net consistently achieved higher and more stable DSC values compared to the 2D nnU‐Net, suggesting improved segmentation performance. Colored vertical dashed lines indicate the epoch at which the highest validation DSC was recorded for each fold.

Figure 3 illustrates the evolution of DSC over training epochs for the four neural network models. For the U‐Net and attU‐Net models (Figure 3a,b), both training (dashed line) and validation (solid line) curves are shown. For the 2D U‐Net and attU‐Net models (Figure 3a,b), both training (dashed lines) and validation (solid lines) curves are shown. Validation performance improved rapidly during the early phase of training and began to stabilize within the first ~20 epochs. The highest validation Dice score was achieved at epoch 27 for U‐Net and epoch 45 for attU‐Net, after which validation performance remained stable without further meaningful improvement. Although training Dice continued to increase slightly beyond these points, the widening gap between training and validation curves indicated the onset of limited overfitting. Final model weights were therefore selected at the epochs corresponding to peak validation performance rather than at the final epoch. For the 2D and 3D nnU‐Net models (Figure 3c,d), the mean validation dice score across 5 cross‐validation folds is shown, with shaded regions representing ± standard deviation. Both models exhibited rapid improvement within the first ~30 epochs, after which the curves plateaued and remained stable for the rest of training. The shaded region demonstrates consistent performance across folds with limited variability. These results suggest that nnU‐Net architectures converge quickly and maintain stable generalization, with little to gain from extending the training effort beyond 1000 epochs. To summarize, the 3D nnU‐Net achieved the highest DSC performance overall, followed by the 2D nnU‐Net, attU‐Net, and U‐Net respectively.

To ensure a fair comparison, the 2D U‐Net was trained for 100 epochs, matching the training duration of both nnU‐Net variants. However, its validation DSC plateaued at 0.62 around epoch 50 and showed no further improvement, confirming that the limited segmentation accuracy was due to architectural constraints rather than insufficient training. In contrast, the 2D nnU‐Net achieved a DSC of 0.74, while the 3D nnU‐Net outperformed all models with a DSC of 0.81.

**FIGURE 4:**
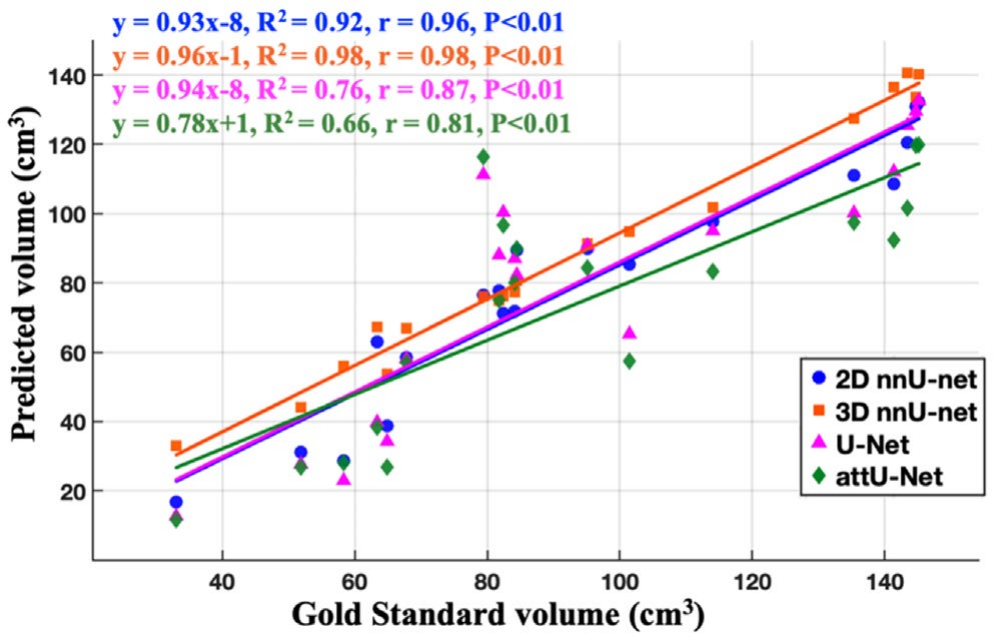
Comparison of predicted vs gold standard volumes across neural network models. Scatter plots illustrating the relationship between predicted and gold standard volumes (in cm^3^) for the four segmentation models: 2D nnU‐Net, 3D nnU‐Net, U‐Net and attU‐Net. Here, gold standard refers to manually annotated placentome volumes. Each model is represented by distinct markers and colors. Regression lines and equations are shown for each model, along with corresponding coefficients of determination (*R* ^2^), Pearson correlation coefficients (*r*), and significance levels (P).

**FIGURE 5:**
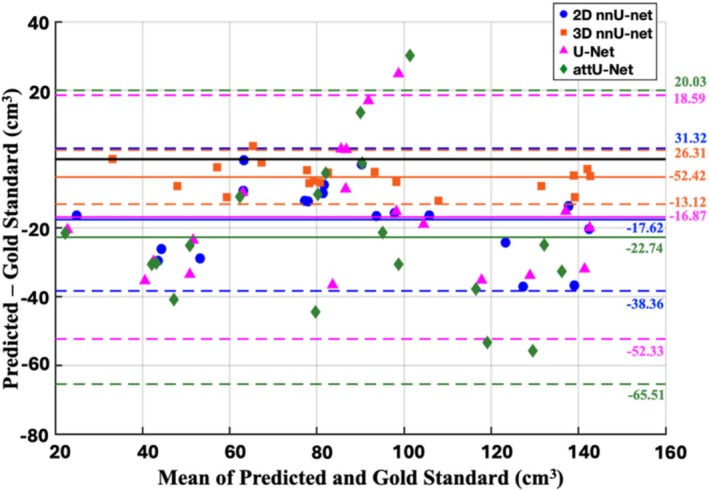
Bland–Altman analysis of volume prediction accuracy across different network configurations. Bland–Altman plots showing the agreement between gold standard (manual segmentation) and predicted placentome volumes for four deep learning models (2D nnU‐Net, 3D nnU‐Net, U‐Net and attU‐Net). Each point represents one test case, with the x‐axis showing the mean of gold standard and predicted volumes and y‐axis showing the difference (predicted − gold standard).

Volume estimation accuracy was further assessed by comparing the computationally predicted volumes with the gold standard manual volumes (Figure 4). Strong correlations were observed for the 3D nnU‐Net (*R* ^2^ = 0.96, *r* = 0.98, *p* < 0.01) and 2D nnU‐Net (*R* ^2^ = 0.92, *r* = 0.96, *p* < 0.01), indicating high consistency between predicted and true volumes. U‐Net and AttU‐Net showed lower agreement, with *R* ^2^ values of 0.78 and 0.66, respectively. These results confirm the superior performance of the 3D nnU‐Net in volume estimation, with the 2D nnU‐Net also providing robust predictions, while U‐Net and AttU‐Net were less reliable. Bland–Altman analysis (Figure 5) demonstrated that 3D nnU‐Net predictions had the narrowest limits of agreement and minimal bias compared to other models, indicating superior reliability in volume estimation. In contrast, U‐Net and AttU‐Net exhibited wider variability, with AttU‐Net in particular showing systematic underestimation at larger volumes.

**FIGURE 6:**
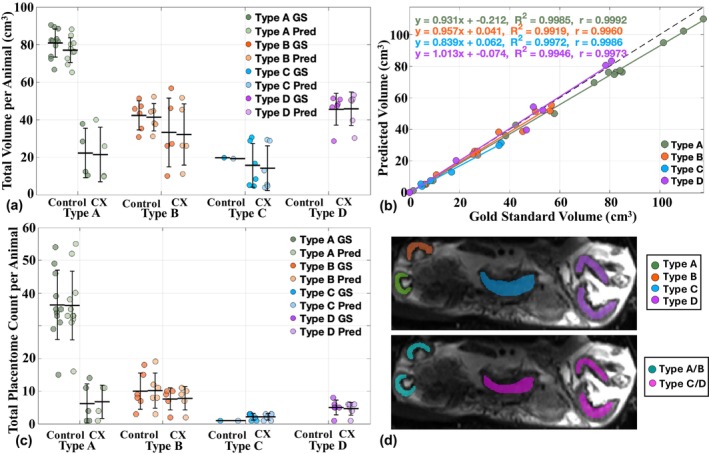
Comparison of gold standard and predicted placentome volumes across morphological types and experimental conditions, using the 3D nnU‐Net model. (a) Total placentome volume per animal for each placentome type (a–d), shown separately for Control and carunclectomised (CX) pregnancies. Each dot represents one animal's total placentome volume for the given type. Horizontal lines indicate group means, and vertical bars represent standard deviation (SD). Bold‐colored markers correspond to manual ground truth (GT) segmentations, while faded‐colored markers represent automatic segmentations generated by a 3D nnU‐Net model. (b) Scatter plot of predicted versus gold standard volumes, color‐coded by morphological type. Each data point corresponds to a single animal from the validation set, with total placentome volume aggregated per type. Dashed line (y = x) represents ideal fitting. (c) Total placentome count per animal for each placentome type, grouped and color coded as in (a). Each dot reflects the number of placentomes detected in a single animal. Mean ± SD are overlaid as horizontal lines and vertical bars, respectively. (d) Representative MR images showing gold standard (top) and automated (bottom) placentome segmentation and classification, generated using the 3D nnU‐Net and YOLOv11x models, with placentomes color‐coded by morphological type.

Overall, the 3D nnU‐Net outperformed all other models in both segmentation accuracy and volume estimation reliability, while AttU‐Net provided incremental improvement over the conventional U‐Net but remained inferior to nnU‐Net approaches. The 3D nnU‐Net model demonstrated strong agreement between predicted and gold standard placentome volumes across all morphological types and groups. Predicted volumes closely matched manual segmentations in both Control and CX pregnancies, with minimal bias and high correlation (Figure 6a). Regression analysis (Figure 6b) showed near‐unity slopes and coefficients of determination (*R* ^2^ >0.99) for all placentome types, confirming excellent volumetric accuracy and consistency. These results indicate that the network effectively generalized across physiological conditions and the morphological diversity of placentomes.

Beyond segmentation accuracy, analysis of placentome morphology revealed clear volumetric distinctions between types and groups, as reported in Table 2. All volumetric measurements were derived exclusively from voxel‐wise 3D nnU‐Net segmentation outputs. Type A placentomes contributed the largest total volume in Control animals, whereas CX pregnancies showed a lower total volume for Type A and a higher proportion of Type B–D. The two‐way repeated‐measures ANOVA (conducted on Types A and B) revealed a significant main effect of placentome Type (*F* = 50.82, *p* = 0.0002), with type B placentomes having significantly larger volumes than type A across both groups. A significant interaction between Group and Type was also observed (*F* = 19.78, *p* < 0.001), suggesting that the effect of placentome type on volume differed between Control and CX groups. No significant main effect of group was detected (*F* = 0.18, *p* = 0.675), indicating that overall placentome volume did not differ between physiological groups. Together these findings suggest that placentome type, rather than physiological group, is the primary determinant of mean volume differences.

**TABLE 2:** Summary of placentome volumes across morphological types and experimental groups, based on gold standard and predicted measurements. Predicted volumes were generated using the 3D nnU‐Net model, which outperformed alternative architectures in accuracy. Values include total volume, mean volume ± standard deviation (SD), and placentome count.

Type	Group	Gold standard				Predicted			
		Total vol. (cm^3^)	Mean vol. ± SD (cm^3^)	Placentomes count	Number of animals	Total vol. (cm^3^)	Mean vol. ± SD (cm^3^)	Placentomes count	Number of animals
A	Control	863.89	2.22 ± 0.47	390	11	817.72	2.11 ± 0.37	387	11
A	CX	76.07	2.11 ± 0.41	36	5	71.79	2.24 ± 0.52	32	5
B	Control	253.72	4.53 ± 1.15	56	6	248.36	4.21 ± 1.03	59	6
B	CX	166.13	4.37 ± 2.19	38	5	160.50	41.15 ± 2.14	39	5
C	Control	19.76	6.59 ± 0.37	3	1	19.29	6.43 ± 0.49	3	1
C	CX	97.78	7.52 ± 3.36	13	6	83.42	7.58 ± 3.00	11	6
D	Control	‐	‐	0	0	‐	‐	0	0
D	CX	273.47	9.43 ± 2.19	29	6	274.49	9.81 ± 2.25	28	6

### 3.2 Placentome classification

The placentome detection model evaluation across a range of Intersection over Union (IoU) thresholds (0.75–0.90) and confidence thresholds (0.5–0.8) identified IoU = 0.75 with confidence = 0.5 as the optimal configuration (Table 3). At IoU thresholds <0.75, which are not reported in the table, the model frequently produced multiple overlapping detections for placentomes located in close proximity, making it difficult to distinguish individual instances reliably. Confidence thresholds lower than 0.5 were likewise excluded, as they often resulted in the same placentome being assigned to both classes, reflecting unstable predictions. Raising the threshold from the conventional 0.5–0.75 ensured that only well‐localized predictions contributed to detection metrics, providing a more conservative measure of spatial accuracy. This configuration achieved a precision of 0.8871, recall of 0.9209, F1 score of 0.9037, and mAP50 of 0.9511. At higher IoU thresholds, the stricter criterion became overly restrictive, discarding well‐aligned detections and reducing measured accuracy. Using this optimal setting, performance was then assessed per placentome type and overall (Table 4).

**TABLE 3:** Performance metrics (precision, recall, F1, mAP50) of the placentome detection model across combinations of confidence thresholds and intersection over union (IoU) values. The row with boldface numbers (Conf = 0.5, IoU = 0.75) indicates the configuration that achieved the best overall performance, with the highest mAP50 and F1‐score, providing the optimal balance between precision and recall.

Conf	IoU	Precision	Recall	F1	mAP50
**0.5**	**0.75**	**0.8871**	**0.9209**	**0.9037**	**0.9511**
0.5	0.8	0.8776	0.9094	0.8932	0.949
0.5	0.85	0.864	0.9082	0.8855	0.9438
0.5	0.9	0.822	0.8958	0.8573	0.9324
0.6	0.75	0.8871	0.9209	0.9037	0.9505
0.6	0.8	0.8776	0.9094	0.8932	0.9488
0.6	0.85	0.864	0.9082	0.8855	0.9446
0.6	0.9	0.822	0.8958	0.8573	0.9336
0.7	0.75	0.8871	0.9209	0.9037	0.9446
0.7	0.8	0.8776	0.9094	0.8932	0.9432
0.7	0.85	0.864	0.9082	0.8855	0.9402
0.7	0.9	0.822	0.8958	0.8573	0.9316
0.8	0.75	0.8988	0.9089	0.8997	0.9303
0.8	0.8	0.8787	0.9089	0.8935	0.929
0.8	0.85	0.8627	0.9089	0.8852	0.9265
0.8	0.9	0.8172	0.9042	0.8585	0.9188

**TABLE 4:** Performance metrics of the object detection model (YOLOv11‐X) evaluated on 2D MRI slices. Results are reported per placentome type and overall, including precision, recall, F1 score, mean average precision at IoU threshold 0.5 (mAP50), and averaged across IoU thresholds from 0.5 to 0.95 (mAP50‐95).

Placentome type	MRI slices	Number of placentomes	Precision	Recall	F1	mAP50	mAP50‐95
AB	317	1478	0.988	0.868	0.924	0.956	0.932
CD	109	262	0.786	0.973	0.870	0.946	0.937
Overall	442	1740	0.887	0.921	0.904	0.951	0.935

As reported in Table 4, the YOLOv11x object detection model demonstrated strong performance on the placentome dataset across all evaluated metrics. The dataset included a higher number of type AB placentomes (*n* = 1478) compared to type CD (*n* = 262), which may have influenced model performance due to class imbalance. For type AB, the model achieved exceptionally high precision (0.988), indicating accurate detection with minimal false positives. However, recall was moderately lower (0.868), suggesting that some true AB placentomes were missed. In contrast, for the less frequent type of CD, the model exhibited excellent recall (0.973), successfully identifying most instances, but with a lower precision (0.786), reflecting more frequent overlap with the AB category. Some of these apparent misclassifications likely reflect transitional placentome morphologies with visual features intermediate between AB and CD, rather than clear detection errors. Despite these differences, both classes showed strong localization performance, as evidenced by high mAP50 (0.956 for AB and 0.946 for CD) and mAP50–95 (0.932 and 0.937, respectively). Overall, the model achieved balanced and robust detection across placentome types (precision: 0.887, recall: 0.921, mAP50: 0.951, mAP50–95: 0.935), supporting its effectiveness for automated placentome analysis, even in the presence of class imbalance.

**FIGURE 7:**
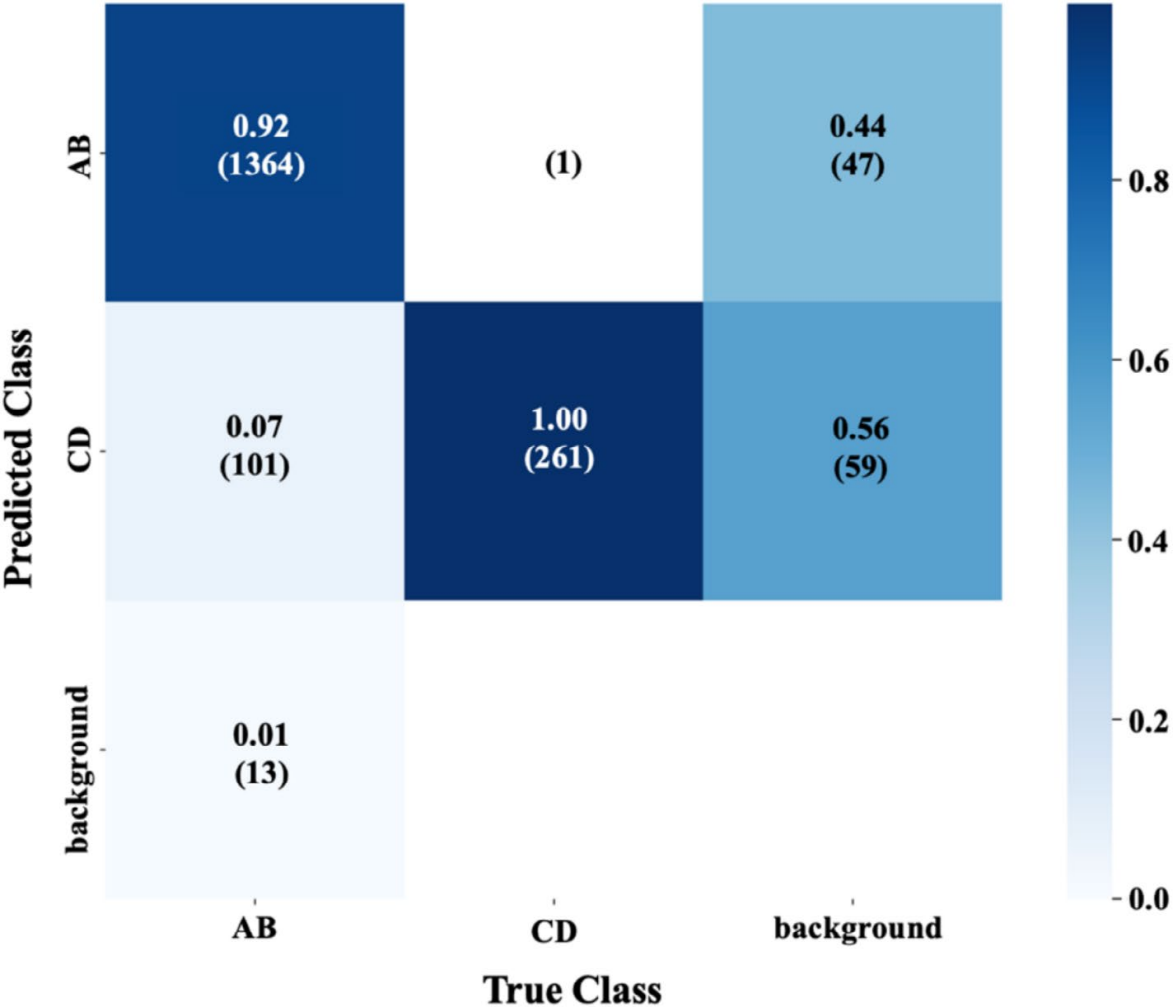
Normalized confusion matrix for YOLOv11x placentome detection. Rows correspond to the predicted class and columns to the true class (AB placentomes, CD placentomes, and background/no‐detection). Each column is normalized to sum to 1, so the diagonal entries represent per‐class recall: 0.92 of true AB placentomes and 1.00 of true CD placentomes were correctly detected. Off‐diagonals indicate misclassifications (e.g., 0.07 of AB placentomes were predicted as CD) and false negatives (e.g., 0.44 of background regions were spuriously labeled as AB). Numbers in parentheses show the corresponding absolute counts of placentomes in each cell.

The model correctly identified 92% (1364/1478) of all AB placentomes and achieved near‐perfect recall 99.6% (261 out of 262) for CD placentomes (Figure 7). Off‐diagonal entries reflect misclassification patterns: among true AB placentomes, 7% (101 out of 1478) were incorrectly labeled as CD, and 1% (13/1478) were missed entirely and classified as background. The background column, representing all regions without true placentomes, reveals a notable tendency toward false positives: 44% of background predictions were spuriously labeled AB and 56% as CD. Importantly, these false‐positive detections did not influence the placentome volume estimates reported in Table 2. Overall, the results highlight the model's strong performance in detecting CD placentomes and demonstrate its high accuracy in identifying AB placentomes, correctly detecting 1364 out of 1478 instances (92%). However, the confusion matrix also shows that false positives in background regions remain difficult to eliminate, as some non‐placentome areas are occasionally misclassified as placentomes.

## 4 DISCUSSION

In this study, we evaluated the performance of deep learning models U‐Net, attU‐Net, 2D nnU‐Net, and 3D nnU‐Net for automated segmentation of placentomes in sheep MR images. The results show significant performance improvements with self‐configuring nnU‐Net architectures. The 3D nnU‐Net achieved a mean Dice score of 0.81, which was higher than the 2D nnU‐Net at 0.74 and the conventional U‐Net baseline at 0.62 and attU‐Net at 0.63. These findings highlight the architectural limitations of traditional U‐Net models and highlight the adaptability of the nnU‐Net framework in tailoring preprocessing techniques, intelligent patching strategies such as optimized selection and sizing of image sub‐volumes, and network parameters to the specific challenges of placentome segmentation in ovine MRI scans.

Among the evaluated models, 3D nnU‐Net demonstrated superior segmentation accuracy, likely due to its ability to leverage volumetric context, which improved boundary fidelity, reduced volumetric bias, and preserved anatomical continuity across slices. These advantages support its effectiveness for anatomically complex, multi‐placentome structures. Manual annotation of placentomes required approximately 25–30 min per case, reflecting the substantial time burden of delineating multiple structures across a 4D MRI acquisition. In contrast, the automated pipeline generated complete 3D segmentation masks from each 4D MRI stack in ~10 s, representing a reduction in analysis time of more than 95%. This improvement in efficiency enables high‐throughput processing of large or longitudinal datasets and enhances reproducibility by removing operator‐dependent variability. Beyond segmentation accuracy, the volumetric outputs reflected expected physiological adaptations following carunclectomy. CX pregnancies exhibited a lower total placentome count and volume compared to controls, consistent with the reduced caruncular surface area available for placentation (Flouri et al., 2021). The 3D nnU‐Net model accurately captured these group‐wise differences, indicating its sensitivity to biologically meaningful variations rather than mere geometric features. Importantly, these findings demonstrate that automated segmentation can preserve physiological interpretability – linking structural changes in placentome number and volume to underlying maternal‐fetal interface adaptations. Qualitative analysis revealed that the 3D nnU‐Net mitigates failure modes common to 2D networks, including false positives in background regions and under‐segmentation of small, low‐contrast placentomes. While the 2D nnU‐Net successfully recovered substantially more placentomes than the baseline, it exhibited slice‐by‐slice inconsistencies and occasional misclassifications at image borders, indicating the limitations of 2D modeling for anatomically heterogeneous structures.

Importantly, improvements in Dice score were accompanied by substantial reductions in boundary error as measured by HD95. The 3D nnU‐Net achieved the lowest HD95 (1.89 mm), compared to 3.75 mm for the 2D nnU‐Net and >12 mm for the U‐Net and attU‐Net baselines. This indicates not only improved volumetric overlap but also markedly superior boundary fidelity, which is critical for accurate quantification of small, spatially discrete placentomes. Lower HD95 values reflect reduced extreme surface deviations and improved anatomical plausibility of the predicted placentome boundaries, reinforcing the advantage of volumetric context in 3D architectures.

These qualitative findings align with the quantitative evaluation (Figure 3), where the 3D nnU‐Net achieved the highest Dice scores and the lowest variability across folds. Its ability to detect subtle or morphologically atypical placentomes is particularly important for volumetric and morphological assessments, suggesting that 3D nnU‐Net may provide a more reliable and reproducible tool for automatic placentome segmentation compared to 2D models.

Our findings align with previous work in human placental MRI, where 3D convolutional neural networks consistently outperform their 2D counterparts in both accuracy and robustness (Han et al., 2019; Huang et al., 2023; Shahedi et al., 2020; Shahedi et al., 2021). However, direct translation of human placenta segmentation techniques to the ovine placenta presents distinct challenges. Unlike the centralized discoid morphology of human placentas, ovine pregnancies comprise 60–80 discrete placentomes per animal, each varying in shape, size, and orientation. This anatomical heterogeneity complicates both manual annotation and automated segmentation, particularly for marginally located or morphologically atypical placentomes.

In parallel, recent transformer‐based and hybrid convolution–transformer architectures, such as UNETR (Hatamizadeh et al. 2022), Swin‐UNETR (Hatamizadeh et al. 2022), and nnFormer (Zhou et al. 2023), have demonstrated strong performance in large‐scale 3D medical image segmentation tasks, including volumetric MRI. These models leverage long‐range self‐attention to capture global contextual information and have achieved state‐of‐the‐art results in multi‐organ and neuroimaging benchmarks. However, such approaches typically require substantially larger annotated datasets to achieve stable training and robust generalization. Given the limited size of preclinical ovine placental MRI datasets and the highly discrete, spatially heterogeneous nature of placentomes, the present study focused on U‐Net–derived volumetric architectures, including the self‐configuring nnU‐Net framework, which is well suited to moderate‐sized datasets. Systematic evaluation of transformer‐based and hybrid architectures represents an important direction for future work as larger, multi‐centre placental MRI datasets become available.

Model development in preclinical imaging is further constrained by limited dataset sizes under the 3Rs (Replace, Refine, Reduce) that restrict animal use and ensure welfare. These constraints hamper generalizability and challenge the deployment of deep learning‐based automation for image quantification. Advanced medical imaging, however, directly advances the 3Rs by enabling extraction of richer quantitative information from fewer animals. High‐resolution longitudinal MRI allows repeated, non‐invasive measurements across gestation, thereby improving statistical power without increasing cohort size. In this study, we demonstrated that volumetric architectures could achieve high segmentation accuracy even in small, heterogeneous datasets, supporting scalable and reproducible quantification in preclinical placental imaging studies.

Although YOLO‐based detectors perform joint object localisation and class prediction, a fully unified segmentation–classification framework was not adopted in this study. YOLO architectures operate on 2D images and produce bounding‐box representations rather than voxel‐wise masks, which limits their ability to capture fine‐grained 3D placentome morphology and the volumetric measurements required for biological interpretation. In contrast, nnU‐Net provides dense 3D segmentation with anatomically consistent boundaries, enabling accurate quantification of placentome volume and number. For this reason, segmentation and classification were treated as complementary but distinct tasks.

Building on this design choice, by integrating a fully automated nnU‐Net segmentation pipeline with a YOLOv11x detection module, this study presents a comprehensive end‐to‐end framework for high‐throughput, quantitative analysis of ovine placentomes. The combined approach addresses the dual challenges of segmenting numerous discrete placentomes and classifying their morphological subtypes within a single workflow. While the immediate application is to sheep pregnancy, the reliability of this pipeline establishes a foundation for broader preclinical studies of cotyledonary (multi‐placentome) placentas. In practice, the proposed automated workflow replaces labour‐intensive, slice‐by‐slice contouring and ad‐hoc labelling, substantially reducing manual work and saving analysis time while standardizing outputs across operators and timepoints. Importantly, the automatic segmentation and classification of placentomes are entirely non‐invasive. Unlike conventional approaches that rely on post‐mortem tissue collection for placentome categorization, this method enables in vivo analysis—supporting animal welfare and facilitating longitudinal monitoring and advancing the principles of the 3Rs by maximizing information yield from fewer animals.

YOLOv11x achieved a recall of 92.3% for AB placentomes (1364/1478) and 99.6% for CD placentomes (261/262). The near‐perfect CD recall likely reflects both their smaller sample size and more consistent morphology, which could have made them easier for the model to learn and recognize. However, CD precision (0.786) and overall mAP@0.5 (0.946) reveal a false‐positive rate that must be balanced against sensitivity. Moreover, the confusion matrix (Figure 3) shows that 7% of AB placentomes, particularly those with unusually large size or atypical shape, may share visual characteristics with CD placentomes, leading to mislabelling. This overlap highlights a limitation in the model's ability to distinguish between placentome types when morphological boundaries are ambiguous. In future work, we aim to validate on larger, more balanced MRI datasets and investigate class‐aware loss weighting, synthetic augmentation, and incorporation of size‐based or spatial‐context features to improve differentiation between AB and CD placentomes, especially in cases where visual cues alone may be insufficient.

Accurate segmentation and classification of placentomes are critical for quantifying placental development and identifying early markers of fetal growth restriction, or other pregnancy outcomes in sheep models. Robust, automated pipelines enable longitudinal tracking of placentome number, size, and morphology across gestation and link these structural changes to functional MRI metrics (e.g., oxygenation, perfusion) (Flouri et al., 2025).

Analysis of placentome volumes revealed physiologically meaningful differences across morphological types and groups. The test set comprised 19 animals, including 11 Controls and 8 CX pregnancies. Of the Controls, 10 were collected at mid‐gestation (109–111 days), and only one at late gestation (139–140 days), while the CX group included 5 mid‐gestation and 3 late‐gestation animals. This gestational distribution likely accounts for the absence of Type D and the limited presence of Type C placentomes in the Control group, as more everted placentome types (C and D) are typically observed later in gestation (Flouri et al., 2021). Carunclectomy has been shown to influence placentome development, promoting a shift toward more everted morphologies (Types C and D) and reducing the prevalence of more concave types (A and B) (Zhang et al., 2016). This may explain the presence of more Type C and D placentomes in CX animals, even at mid‐gestation. In both Control and CX pregnancies, Type B placentomes exhibited larger mean volumes than Type A, consistent with their established role in nutrient transfer and fetal growth support (Vonnahme et al., 2008). Compared to Controls, CX pregnancies showed reduced total placentome volume for Type A and a higher proportion of Types B‐D (see Table 2), reflecting compensatory adaptation to the reduced caruncular surface area following carunclectomy. While volumetric comparisons in Controls were limited to Type A and B due to the low incidence of C and D (only 3 Type C placentomes and no Type D), these findings demonstrate the functional significance of placentome type and highlight the utility of automated segmentation and classification algorithms in detecting and quantifying structural adaptations across gestation. While manual segmentation has traditionally been used to extract biologically relevant information, the automated approach proposed in this study produces segmentation results that align closely with manual annotations with the added benefits of reduced labour, improved reproducibility, and suitability for large‐scale analyses.

These volumetric findings are further supported by comparisons with post‐mortem placentome weights reported in the literature. Placentome volumes estimated from MRI, using both expert manual segmentation (gold standard) and automated segmentation, were converted to weight using a tissue density of 1.05 g/cm^3^ (Duck, 1990). The resulting average weight ranges were 2.2–2.4 g for Type A, 4.4–4.8 g for Type B, 6.9–8.0 g for Type C, and 9.8–10.6 g for Type D. These estimates reflect the fact that most animals in our study were scanned at mid‐gestation (109–111 days), particularly the control group, which predominantly exhibited Type A and B placentomes, a stage when placentome development is still actively progressing. In contrast, literature‐reported post‐mortem weights, obtained from late‐gestation animals (130–140 days), range from 4.0 to 6.0 g for Type A, 6.0–8.0 g for Type B, 8.5–11.0 g for Type C, and 10.0–12.5 g for Type D (van der Linden et al., 2013; Vonnahme et al., 2008; Zhang et al., 2016). The alignment in relative weight trends across types supports the biological plausibility of our MRI‐derived estimates and highlights the utility of non‐invasive imaging for tracking placental development across gestation.

Several limitations of this study should be acknowledged. First, although the dataset scanned three gestational ages (80, 109–111, and 139–121 days), the relatively small sample size at early and late pregnancy stages may limit generalizability of the deep learning models, which typically benefit from larger and more balanced training data across gestation. Second, manual segmentations used as gold standard may introduce some degree of subjectivity, potentially influencing model performance assessment. In a subset of cases, the automated 3D nnU‐Net correctly identified placentomes that were initially missed in the manual labels; however, it also occasionally produced false positives in regions where anatomical structures such as fetal limbs resembled placentome morphology. Future work will refine the automatic algorithm to reduce these misclassifications and further improve robustness by incorporating additional training examples of confounding structures and exploring post‐processing filters or anatomical priors to suppress biologically implausible detections. Third, in the test set used for morphological analysis (Table 2 and Figure 6a–b), Control animals had no Type D placentomes and only very few Type C placentomes. Consequently, volumetric comparisons and statistical analyses for Types C and D between Control and CX pregnancies could not be performed, and the analysis was limited to Types A and B. Future research will aim to expand the dataset to include a broader range of gestational stages and pathological pregnancies, enabling more precise evaluation of model performance across gestational age and its applicability to abnormal placental conditions. Integrating functional imaging parameters such as perfusion and oxygenation could also provide a more comprehensive assessment of placental health. Another limitation is that all data were acquired using a single MRI protocol at a single institution. While this ensures consistency within our dataset, it may restrict the generalizability of the models to different imaging settings. Although ovine MRI is currently performed only by a limited number of research centres worldwide, future studies could still evaluate robustness under varying acquisition conditions and scanner platforms through collaborative datasets where available. Generalizability could also be further improved by incorporating multi‐centre datasets, applying advanced data augmentation strategies, and leveraging domain adaptation techniques to account for variations in scanner platforms or acquisition parameters. Notably, the self‐configuring nature of nnU‐Net partially mitigates small dataset limitations by optimizing preprocessing, patch selection, and network parameters for the specific data, contributing to stable performance despite these constraints.

In summary, this study demonstrates the efficacy of deep learning models, particularly the 3D nnU‐Net and YOLOv11x in automating the segmentation and classification of placentomes in ovine MRI scans. The 3D nnU‐Net outperformed both the 2D nnU‐Net and conventional U‐Net, leveraging volumetric context to achieve superior anatomical fidelity and segmentation accuracy. Meanwhile, YOLOv11x showed promising classification performance, especially for morphologically consistent placentome types, though challenges remain in distinguishing visually ambiguous cases. Together, these models introduce the first deep‐learning framework for quantitative placental analysis in ovine MRI, offering a robust, non‐invasive, and scalable approach for assessing placental morphology and adaptation. This work enables in vivo quantification of placentome morphology without manual annotation, marking an important step toward automated, high‐throughput analysis in preclinical placental imaging. Despite limitations in dataset size and annotation variability, the results underscore the transformative potential of self‐configuring, volumetric deep learning architectures in advancing preclinical imaging. Future work will focus on expanding dataset diversity, refining classification accuracy by transitioning from the current AB/CD grouping to four‐class (A–D) placentome categorization, which may improve subtype differentiation and biological interpretability. In addition, integrating functional imaging metrics such as perfusion and oxygenation will enable more comprehensive assessments of placental development and health.

## AUTHOR CONTRIBUTIONS

D.F., A.D., J.L.M., A.M. were responsible for the conception and design of the study. D.F., G.A., J.R.T.D, S.L.H., G.W., A.D., J.L.M., A.M. were involved in the acquisition, analysis, and interpretation of data for the work. D.F. drafted the article. All authors contributed to and approved the final version of the manuscript. All authors have read and approved the final version of this manuscript and agree to be accountable for all aspects of the work, ensuring that questions related to the accuracy or integrity of any part of the work are appropriately investigated and resolved. All persons designated as authors qualify for authorship, and all those who qualify for authorship are listed.

## FUNDING INFORMATION

D.F. was supported by Marie Sklodowska‐Curie Postdoctoral Fellowship (101108945—InSilicoPlacenta). The animal work was supported by the Australian Research Council (DP190102263 and DP220103289) and BBSRC (BB/Y514214/1). JLM was funded by an ARC Future Fellowship (Level 3; FT170100431) and a NHMRC Investigator Leader 2 (GNT2041967). A.M. and A.L.D. were supported by BBSRC (BB/Y514214/1), the MRC (MR/X010007/1) and the NIH (R01 HD108833). A.L.D. is part supported by the National Institute for Health and Care Research University College London Hospitals Biomedical Research Centre. JRTD was supported by a University of South Australia Enterprise Postdoctoral Fellowship.

## CONFLICT OF INTEREST STATEMENT

The authors declare no competing interests.

## DATA AVALIABILITY STATEMENT

All data supporting the results are presented in the manuscript. MRI images can be accessed upon reasonable request to the senior authors. The code used for automatic segmentation and classification of sheep placentomes is openly available at https://github.com/georgeadamidess/placentomes.

## Supporting information


**Table S1.** Summary of prior MRI‐based placental segmentation studies. All studies to date have been conducted on human placentas; to our knowledge, no MRI‐based deep learning segmentation studies have been reported for ovine placentomes. Reported metrics are reproduced from the original publications. Imaging protocols, architectures, evaluation metrics, and performance values are reproduced from the original publications.
