# Supplementary material for: A deep learning algorithm for automatic 3D segmentation and classification of the sheep placenta in magnetic resonance images
Source: Physiol Rep. 2026 Apr 17;14(8):e70869. doi: 10.14814/phy2.70869 (PMC13090531; doi:10.14814/phy2.70869)
Supplement: Supplementary file 1 — Table S1. Summary of prior MRI‐based placental segmentation studies. All studies to date have been conducted on human placentas; to our knowledge, no MRI‐based deep learning segmentation studies have been reported for ovine placentomes. Reported metrics are reproduced from the original publications. Imaging protocols, architectures, evaluation metrics, and performance values are reproduced from the original publications. [file PHY2-14-e70869-s001.docx]

**SUPPORTING INFORMATION**

**Table S1.** Summary of prior MRI-based placental segmentation studies. All studies to date have been conducted on **human placentas**; to our knowledge, **no MRI-based deep learning segmentation studies have been reported for ovine placentomes.** Reported metrics are reproduced from the original publications. Imaging protocols, architectures, evaluation metrics, and performance values are reproduced from the original publications.

| **Study** | **Segmentation Method** | **MRI Modality / Resolution** | **Dataset Size** | **Model Architecture** | **Evaluation Metrics** | **Reported Performance** |
| --- | --- | --- | --- | --- | --- | --- |
| Wang et al., 2016 (Slic-Seg) | Semi-automatic | T2-weighted MRI; axial 512×448, 0.74 mm, 3mm; sagittal 256×256,1.48 mm, 4mm | 16 fetuses; 32 volumes (axial+sagittal) | Online Random Forest + CRF + 4D Graph Cuts refinement | Dice, ASSD | **Axial:** Dice **0.89 ± 0.02**, ASSD **1.89 ± 0.39 mm; Sagittal:** Dice **0.88 ± 0.02**, ASSD **1.99 ± 0.38 mm** |
| Wang et al., 2018 (DeepIGeoS) | Semi-automatic | Axial T2‑weighted SSFSE; 0.74–1.58 mm in‑plane; 3–4 mm slice; resampled to 1×1 mm | 25 MRI volumes | 2D P‑Net + 2D R‑Net + CRF-Net | Dice, HD | Dice 0.92; HD 6.4 mm |
| Alansary et al., 2016 | Fully automatic | T2-weighted Single Shot Fast Spin Echo; multiple orthogonal stacks; 1.25×1.25×2.5 mm and 0.84×0.84×4 mm | 66 subjects (44 healthy; 22 healthy/IUGR) | 3D multi-scale CNN + 3D dense CRF | Dice, AHD | Dice 0.57–0.72; AHD 4.9–8.4 mm (three experimental settings) |
| Shahedi et al., 2020 | Fully automatic | 3D T2-weighted fetal MRI; axial; 1.0547–1.7188 mm in-plane; 7.0 mm slice thickness | 100 MRI volumes | Modified 3D U-Net | Dice, ΔV | Placenta: Dice 0.82±0.061, ΔV −53±100 cm³ |
| Shahedi et al., 2021 | Fully automatic | 3D T2-weighted fetal MRI; axial; Normal pregnancies: 1.05–1.72 mm in-plane, 7 mm slice thickness; Pregnancies with suspected placental abnormalities: 1.17–1.76 mm in-plane, 7 mm slice thickness | 199 MRI volumes (100 normal pregnancies; 99 suspected placenta accreta spectrum (PAS) and other abnormalities) | 3D U-Net | Dice, ΔV | Normal pregnancies: Dice 0.82±0.061, ΔV -53±100 cm^3^; Mixed pregnancies: Dice 0.83±0.058, ΔV -12±151 cm^3^ |
| Shahedi et al., 2022 | Fully automatic | 3D T2-weighted fetal MRI; axial; 1.05–1.95 mm in-plane; 7 mm slice thickness | 241 MRI volumes | 3D U-Net | Dice, ΔV | Dice 0.80±0.10, ΔV 20±198 cm³ (0.11±0.23) |
| Liu et al., 2023 | Fully automatic | T2-weighted HASTE MRI; 0.976×0.976 mm in-plane; 5 mm slice thickness | 154 women underwent MRI scans at both 14–18 weeks of gestation & 19–24 weeks | SADL (CC + FPA modules) | Dice | Dice 0.83±0.06 (14–18 wks), 0.84±0.05 (19–24 wks) |
| Huang et al., 2023 | Fully automatic | Axial & sagittal T2-weighted MRI; axial: 1.05–1.95 mm, 7 mm; sagittal: 1.17–1.76 mm, 5 mm | 244 axial volumes; 101 sagittal volumes | 3D U-Net 3+ | Dice, HD, ΔV | Sagittal: Dice 0.88±0.031, HD 20.6±10.6 mm, ΔV -16.6±109; Axial: Dice 0.83±0.051, HD 19.3±9.2 mm, ΔV −11.2±17.9 |
| Li et al., 2023 | Fully automatic | FIESTA MRI (axial, sagittal, coronal); matrix 224×224; slice thickness 5–7 mm; in-plane pixel spacing not reported | 200 MRI volumes (103 normal pregnancies, 97 pregnancies with PAS) | RFU-Net (ResNet34 + FMF + RSM) | MIoU, Dice, HD, Accuracy | MIoU 0.86, Dice 0.93, HD 4.31, Accuracy 0.999 |

MIoU, mean intersection over union; Dice, Dice similarity coefficient; ASSD, average symmetric surface distance; HD, Hausdorff distance; ΔV, volume difference; AVD, absolute volume difference; AHD, average Hausdorff distance; CNN, convolutional neural network; CRF, conditional random field; FCNN, fully convolutional neural network; SADL, spatial attentive deep learning; CC, criss‑cross attention; FPA, feature pyramid attention; RFU‑Net, refinement fusion U‑Net; ResNet‑34, 34‑layer residual network; FMF, fusion multiscale feature module; RSM, refinement segmentation module.
